# Effects of repetitive peripheral magnetic stimulation *vs*. conventional therapy in the management of carpal tunnel syndrome: a pilot randomized controlled trial

**DOI:** 10.7717/peerj.15398

**Published:** 2023-05-18

**Authors:** Arachaphon Panathoop, Jittima Saengsuwan, Ratana Vichiansiri

**Affiliations:** Department of Rehabilitation Medicine, Faculty of Medicine, Khon Kaen University, Khon Kaen, Thailand

**Keywords:** Carpal tunnel syndrome, Repetitive peripheral magnetic stimulation, Pilot

## Abstract

**Background:**

Carpal tunnel syndrome (CTS) is a prevalent entrapment neuropathy resulting in hand pain, numbness and/or weakness, which significantly impairs hand function in daily activities. Repetitive peripheral magnetic stimulation (rPMS) is a potential therapeutic option for focal peripheral nerve disease and may be beneficial for CTS treatment. We aimed to compare the effects of rPMS and conventional therapy in the management of CTS.

**Methods:**

A blinded assessor randomly assigned 24 participants with electrodiagnostically-confirmed mild or moderate CTS to either rPMS or conventional therapy. Both groups were briefed on disease progression and tendon-gliding exercises. In the intervention group, the rPMS protocol, five sessions of rPMS—with a frequency of 10 Hz, 10 pulses/train, and 100 trains/session—were performed over a period of 2 weeks, with three sessions in the first week and two sessions in the second week. At baseline and the end of the second week, the Boston Carpal Tunnel Questionnaire, pinch strength, and electrodiagnostic results were evaluated.

**Results:**

The rPMS group demonstrated significantly greater within-group improvement in symptom severity scores (2.3 *vs*. 1.6, *p* = 0.009) and pinch strength (10.6 lbs *vs*. 13.8 lbs, *p* < 0.001). Regarding electrodiagnostic parameters, sensory nerve action potential (SNAP) amplitude was significantly increased (8.7 µV *vs*. 14.3 µV, *p* = 0.002) within the group treated with rPMS. With conventional therapy, there were no statistically significant within-group differences. Multiple linear regression models showed that there were no significant differences in other outcomes in between-group comparisons.

**Conclusions:**

Five sessions of rPMS resulted in significant reduction in symptom severity, improvement in pinch strength and increase in SNAP amplitude. Future research should investigate the clinical utility of rPMS using a larger sample and longer treatment and follow-up durations.

## Introduction

Carpal tunnel syndrome (CTS) is an entrapment neuropathy of the median nerve at the wrist that accounts for 90% of all entrapment neuropathies (Aroori & Spence, 2008; Rosario & De Jesus, 2022). Pain, numbness, weakness, and muscle atrophy are the typical clinical presentations of CTS, which can cause significant hand-use problems affecting daily activities, work functionality, and mental health (Evanoff et al., 2016; Kuo et al., 2014; Shin et al., 2018). The prevalence of CTS in the general population is between 2.7% and 4.9% based on diagnostic criteria, with a higher incidence in females than males, and the most affected age group being between 40 and 60 years (Aroori & Spence, 2008; Atroshi et al., 1999).

Treatment of CTS consists of either surgical or non-surgical management. Generally, surgical treatment is reserved for patients with severe symptoms (Karjalanen et al., 2022). O’Connor, Marshall & Massy-Westropp (2003) found that non-surgical treatments such as oral steroids, wrist splints, ultrasound therapy, laser therapy, and local steroid injection had varying degrees of effectiveness in short and long-term patients with mild to moderate symptoms.

Repetitive peripheral magnetic stimulation (rPMS) is a relatively painless, non-invasive and well-tolerated therapy. rPMS emits magnetic field-induced electrical pulses that stimulate sensorimotor nerve fibers and muscle fiber mechanoreceptors (Kanjanapanang & Chang, 2022). rPMS is a useful tool that can be used in multiple neuromuscular problems, despite the fact that there is limited evidence and the most effective parameters have not yet been demonstrated (Beaulieu & Schneider, 2015). Previous research demonstrated that three to five sessions of rPMS reduce pain in focal peripheral nerve diseases such as entrapment neuropathy, neuroma unresponsive to local steroid injection and brachial plexus injury (Khedr et al., 2012; Leung, Fallah & Shukla, 2014). Regarding CTS, Elena et al. (2016) reported that after 10 daily sessions of rPMS, patients with CTS had increased hand grip strength, reduced symptom severity, and reduced pain of rPMS but did not show significant changes in electrophysiologic outcomes. El Gohary et al. (2015) showed that, compared to a control group, three sessions of rPMS significantly reduced pain in patients with CTS. On the basis of previous studies, we hypothesized that five sessions of rPMS may be sufficient to alleviate CTS symptoms. This study aimed to compare the effects of rPMS and conventional therapy in the management of CTS.

## Materials and Methods

This research was reviewed and approved by the Khon Kaen University Ethics Committee (Ref. HE 621319) and was registered in August 2020 with the Thai Clinical trials registry (TCTR 20200828002). The research was conducted in accordance with the principles of the Helsinki Declaration. The study was an assessor-blinded pilot randomized controlled trial. From September 2020 to May 2021, we enrolled patients with mild to moderate carpal tunnel syndrome who visited our outpatient clinic. Before inclusion, each participant received a thorough explanation of the study protocol and provided written informed consent. Our inclusion criteria were: age at least 18 years and mild to moderate symptom severity. According to electrodiagnostic criteria (Werner & Andary, 2011), a mild degree of severity was characterized by sensory latency of the median nerve greater than 3.5 ms. Sensory and motor latencies of the median nerve are moderately delayed when peak distal sensory latency (DSL) is >3.5 ms and onset distal motor latency (DML) is >4.2 ms. Exclusion criteria included severe cases of CTS defined by small compound muscle action potential (CMAP) amplitude <5 mV or abnormal electromyography in the innervated intrinsic hand muscles, previous surgery for CTS, history of wrist fracture or wrist dislocation, risk and co-morbidity of other neurological diseases (*e.g*., polyneuropathy, cervical radiculopathy, ulnar neuropathy), or contraindications for using rPMS (*i.e*., a cardiac pacemaker or cochlear implant) (Rossi et al., 2009).

### Sample size considerations

For pilot studies, it is recommended that each group contain at least 12 participants (Julious, 2005). Thus, with two groups, a total of 24 participants were recruited.

### Procedures

**Figure 1:**
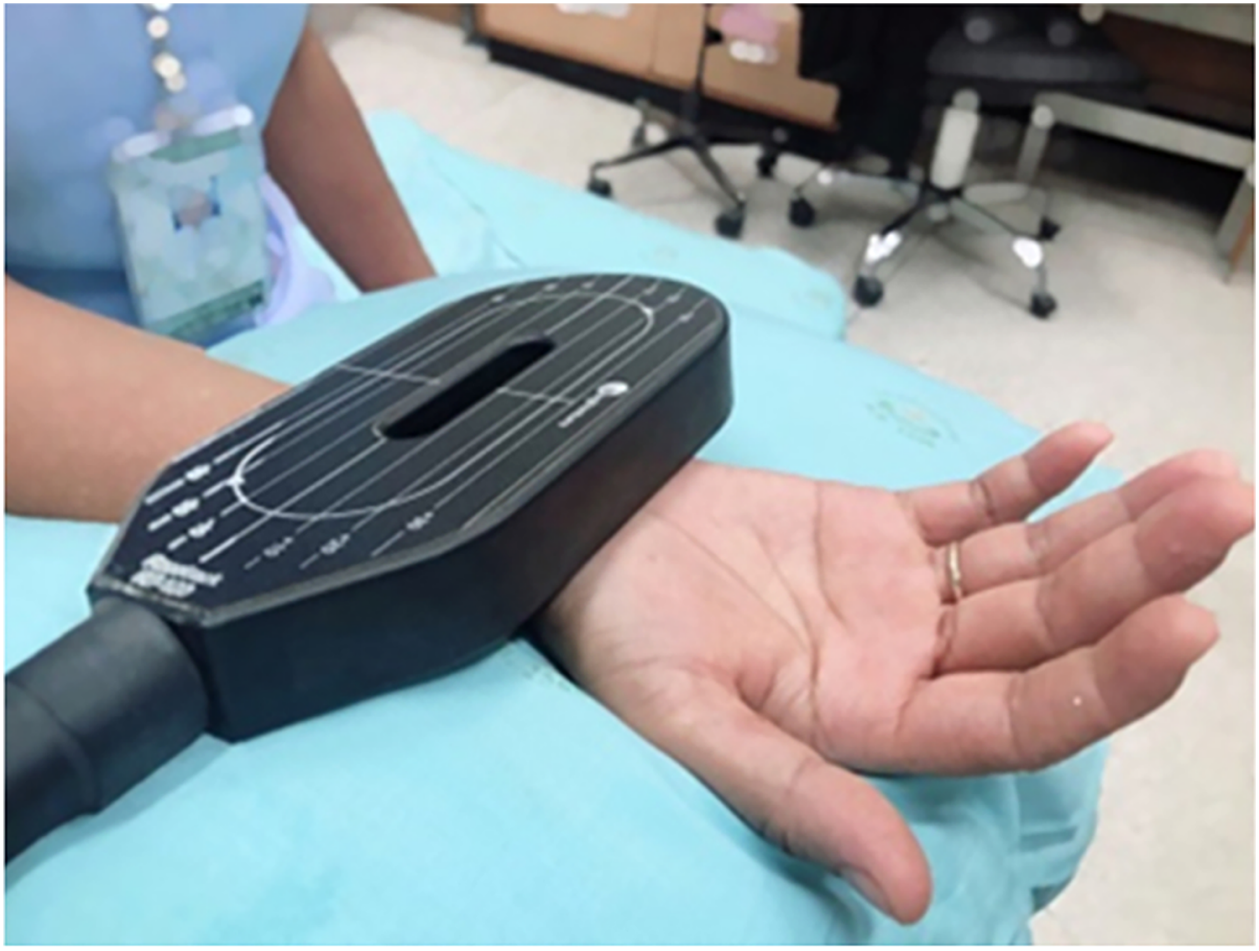
Set-up of rPMS therapy.

Participants were randomly assigned by computer randomization to either conventional therapy or to the rPMS group in a block-of-four design. The nursing assistant, who was not responsible for the allocations, informed the responsible physician about the treatment allocation. The control group (*n* = 12) received conventional therapy, which included patient education, tendon gliding exercise, and vitamins B1-6-12 (Thiamine mononitrate (Vitamin B_1_) 300 mg, Pyridoxine hydrochloride (Vitamin B_6_) 15 mg, Cyanocobalamin (Vitamin B_12_) 150 µg per day). Patients were given pamphlets describing the exercises and giving advice. The information included patient education regarding work modification and ergonomic recommendations. Each exercise began with the wrist and fingers fully extended. During tendon-gliding exercises, the fingers were placed in five discrete positions: straight, hook, tabletop, full fist and straight fist (Rozmaryn et al., 1998). The experimental group (*n* = 12), in addition to the rPMS intervention, also received conventional therapy. The racetrack coil (RT-120; MagVenture, Inc., Alpharetta, GA, USA) was positioned parallel to the median nerve in the middle of the affected wrist. The stimulation coil was fixed with a coil holder, and the coil was in contact with the skin during stimulation (Fig. 1). The stimulus protocol used a frequency of 10 Hz and 10 pulses per train (*i.e*., train duration of 1 s) and an intertrain interval of 5 s. One-hundred trains were applied, leading to a total treatment time of 600 s (10 min). The intensity was 10% above suprathreshold stimulation, which was determined by observing visible hand muscle contraction. Five sessions of magnetic stimulation were performed over a period of 2 weeks, with three sessions in the first week and two sessions in the second week.

### Outcome measures

A blinded assessor performed the outcome measurements. The control and rPMS groups were evaluated at the end of the second week. If patients had bilateral CTS, the intervention and evaluation were performed on the side with the more severe involvement, as determined by electrodiagnosis results.

### Primary outcome

Baseline data regarding CTS symptoms were collected *via* interview using the validated Thai version of the Boston Carpal Tunnel Questionnaire (BCTQ, Upatham & Kumnerddee, 2008), comprising 11 questions pertaining to the symptom severity scale (SSS) and eight questions pertaining to the functional status scale (FSS). Each question was scored using a 5-point Likert scale (with five being the most severe pain or most limited in activity). The SSS and FSS scores are presented using mean values (Levine et al., 1993). The BCTQ is the most frequently used questionnaire for assessing clinical and functional improvement in CTS patients (Atthakomol et al., 2018).

### Secondary outcomes

Hand pinch strength was evaluated using pinch gauges at an elbow flexion of 90 degrees. Three tests were conducted, and the mean was reported in pounds (lbs) (Stegink Jansen et al., 2003).

Median nerve electrodiagnostic measurement was performed at a room temperature of 32 °C using Medtronic Keypoint G4 (Natus Medical Incorporated, Pleasanton, CA, USA). Median motor nerve recording was done using a surface-recording electrode placed on the abductor pollicis brevis (APB) muscle. The stimulating electrode was placed 8 cm from the active electrode at the APB muscle on the median nerve between the palmaris longus tendon, flexor carpi radialis tendon and elbow. We recorded the onset latency and compound muscle action potential (CMAP) amplitude. In addition, antidromic sensory nerve conduction studies evoked at the wrist were recorded from the middle finger: in this case, the stimulating electrode was first positioned 14 cm away from the active electrode which was around the proximal phalanx, and then at the elbow. A minimum of 20 sensory nerve action potentials were averaged. In addition, the peak latency and sensory nerve action potential (SNAP) amplitude were recorded. If the CMAP amplitude was less than 5 mV or there was no response, electromyography (EMG) was performed.

### Statistical analysis

Continuous data are presented by mean and standard deviation for normally distributed data. Median and interquartile ranges are presented in non-normally distributed data. We conducted intention to treat analysis and missing data were addressed using multiple imputation. Paired t-tests were used to compare pre- and post-treatment within-group outcomes. We used a multiple linear regression model to determine difference between groups, with follow-up values as dependent variables (Vickers & Altman, 2001). We adjusted for covariates as follows: baseline value of that variable, severity of CTS determined by EMG criteria and duration after CTS. The statistical significance was set at *p* ≤ 0.05. All statistical analyses were done using Stata version 17.0 (Stata Statistical Software: Release 17. StataCorp LLC, College Station, TX, USA).

## Results

**Figure 2:**
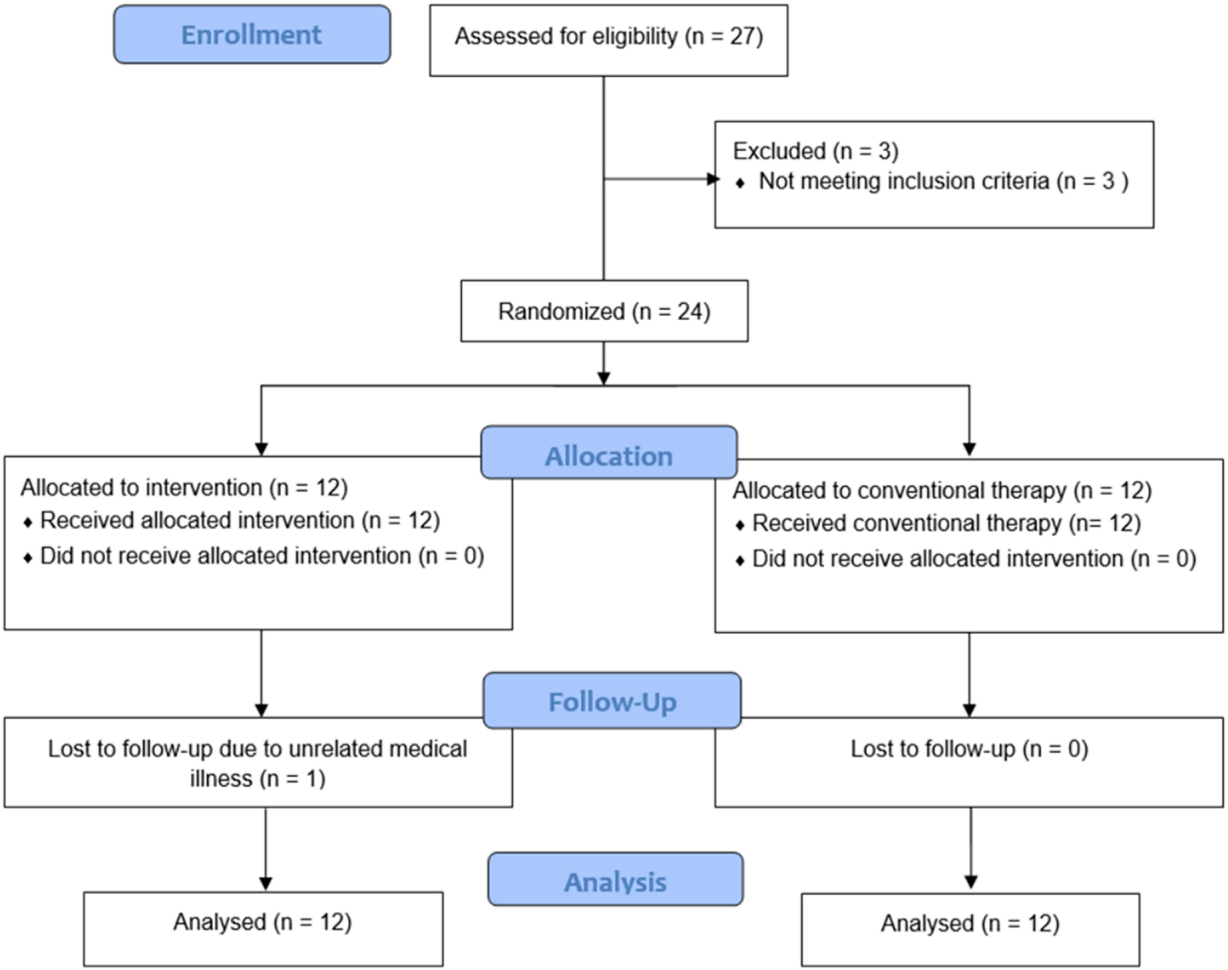
Flow diagram of the study.

Twenty-four participants were enrolled. They were divided into two groups of 12. The rPMS group’s age was 49.7 ± 10.4 years (mean ± standard deviation), and most were female (11/12 = 91.7%). In the control group, the age was 52.3 ± 7.2 years, and eight (66.7%) were female. The respective median duration of working time in the rPMS and control groups was 8 and 7 hours/day. The median duration of CTS symptoms in both groups was 3.5 months (Table 1). One participant in the rPMS group was lost to follow-up due to unrelated medical illness (Fig. 2).

**Table 1:** Baseline characteristics of participants (*n* = 24).

Variable	rPMS (*n* = 12)	Control (*n* = 12)
Age (years), mean (SD)	49.7 (10.4)	52.3 (7.2)
Sex		
- Male	1 (8.3)	4 (33.3)
- Female	11 (91.7)	8 (66.7)
BMI	24.0 (3.8)	24.6 (3.1)
Underlying diseases		
- Hypertension	0 (0)	1 (8.3)
- Dyslipidemia	3 (25.0)	0 (0)
- Others	3 (25.0)	5 (41.7)
Smoking	0 (0)	1 (8.3)
Caffeine	1 (8.3)	0 (0)
Alcohol	1 (8.3)	4 (33.3)
Occupation		
- Government officer	6 (50.0)	6 (50.0)
- Business	4 (33.3)	1 (8.3)
- Agricultural work	0	1 (8.3)
- Others	2 (16.7)	4 (33.3)
Duration of working time (hours/day), median (IQR)	8.0 (5.5–9.8)	7.0 (5.3–8.0)
Symptom duration (months), median (IQR)	3.5 (2.0–6.0)	3.5 (2.0–5.8)
CTS severity		
- Mild	4 (33.3)	4 (33.3)
- Moderate	8 (66.7)	8 (66.7)

**Note:**

Data are reported as *n* (%), unless otherwise indicated. BMI, Body Mass Index; CTS, Carpal Tunnel Syndrome.

Only the rPMS group demonstrated significant improvement in symptom severity scores (SSS) determined by the Thai version of the Boston Questionnaire (2.3 *vs*. 1.6, *p* = 0.009) and pinch strength (10.6 lbs *vs*. 13.8 lbs, *p* < 0.001). As for the nerve conduction study, only rPMS demonstrated a significant within-group increase in the SNAP amplitude of the median nerve (8.7 µV *vs*. 14.3 µV, *p* = 0.002). Multiple linear regression models showed that there was no significant difference when comparing all outcomes between the two groups (Table 2).

**Table 2:** Comparison of clinical and electrodiagnostic outcomes within and between groups.

Outcome	PMS group		Control group		Between group difference^a^	*p*-value^b^
	Mean (SD)	*p*-value^c^	Mean (SD)	*p*-value^c^	Coefficient (95% CI)	
Th-BQ: SSS						
- Pretreatment	2.3 (0.7)		2.1 (0.7)			
- 2 Weeks	1.6 (0.3)	0.009	1.8 (0.4)	0.15	−0.17 [−0.49 to −0.14]	0.27
Th-BQ: FSS						
- Pretreatment	1.8 (0.4)		1.6 (0.7)			
- 2 Weeks	1.5 (0.5)	0.15	1.7 (0.6)	0.80	−0.26 [−0.67 to 0.16]	0.22
Pinch strength (lbs)						
- Pretreatment	10.6 (2.6)		14.6 (4.2)			
- 2 Weeks	13.8 (3.0)	<0.001	15.9 (5.2)	0.11	1.91 [−0.33 to 4.14]	0.09
Peak DSL (ms)						
- Pretreatment	5.1 (1.1)		5.3 (2.7)			
- 2 Weeks	5.0 (1.1)	0.49	5.3 (2.7)	0.82	−0.05 [−0.22 to 0.32]	0.71
SNAP amplitude (µV)						
- Pretreatment	8.7 (4.2)		11.7 (6.2)			
- 2 Weeks	14.3 (5.2)	0.002	15.4 (8.3)	0.12	1.06 [−4.47 to −6.59]	0.69
Onset DML (ms)						
- Pretreatment	4.9 (0.9)		4.6 (1.2)			
- 2 Weeks	4.9 (1.1)	0.12	4.7 (1.1)	0.35	−0.04 [−0.33 to −0.25]	0.78
CMAP amplitude (mV)						
- Pre-treatment	9.2 (2.4)		8.4 (2.8)			
- 2 Weeks	8.3 (1.7)	0.13	8.0 (2.7)	0.35	−0.12 [−1.44 to −1.18]	0.84

**Note:**

^a^Coefficient, corresponding 95% CI and *p*-value^b^ derived from multiple linear regression model. *p*-value^c^ derived from paired t-test (within-group comparison).

When comparing changes in disease severity after treatment, two of the participants in the rPMS group had normal electrodiagnostic results. Only one participant in the control group experienced a change from moderate to mild CTS. There was no statistically significant difference between the two groups in terms of symptom severity change (Table 3).

**Table 3:** Severity of CTS pre and post-treatment in the rPMS and control groups.

	Electrodiagnostic severity					
Group			Post-treatment, *n* (%)			*p*-value*
	Pre-treatment, *n* (%)		Normal	Mild	Moderate	
rPMS	Mild Moderate	4 (33.3) 8 (66.7)	1 (8.3) 1 (8.3)	3 (25.0) 0	0 7 (58.3)	0.18
Control	Mild Moderate	4 (33.3) 8 (66.7)	0 0	4 (33.3) 1 (8.3)	0 7 (58.3)	0.32

**Note:**

* Marginal homogeneity test.

## Discussion

The purpose of the study was to compare the therapeutic effects of five sessions of repetitive Peripheral Magnetic Stimulation (rPMS) *vs*. conventional therapy on electrophysiologic parameters, pinch grip strength, and symptoms of Carpal Tunnel Syndrome (CTS) using the Thai Version of the Boston Questionnaire. Our findings support the therapeutic effect of rPMS, as rPMS improved CTS symptoms, increased finger pinch strength, and enhanced neurophysiologic SNAP amplitude. However, there was no statistically significant positive effect of rPMS when comparing all outcomes between the two groups.

PMS creates a magnetic field that induces an electrical current flow that improves sensory and motor nerve fiber function, which may explain improved symptoms and hand function. rPMS is a novel treatment method and growing evidence suggests that it may be an effective treatment option for treating CTS or other peripheral nerve injuries. rPMS parameters for pain relief and restoration of sensorimotor nerve function vary from 600 to 4,500 pulses (Beaulieu & Schneider, 2015). Low-frequency stimulation of <1 Hz was effective for pain treatment; however, for sensorimotor nerve function restoration, a higher frequency of 10 and 25 Hz appears to be more effective. Additionally, a 10 Hz frequency reduces pain (Beaulieu & Schneider, 2015). As for stimulus intensity, most studies employed suprathreshold stimulation because it is hypothesized that it induces muscle contraction, thereby stimulating proprioception more effectively (Kanjanapanang & Chang, 2022). Therefore, we used 10 Hz stimulation at suprathreshold intensity. We applied a total of 1,000 pulses and a treatment duration of 10 min. The painless stimulation was well received by patients in the treatment group.

In the PMS group, pinch strength was improved from 10.6 to 13.8 lbs. This finding agrees with previous studies. Conforto, Kaelin-Lang & Cohen (2002) and Klaiput & Kitisomprayoonkul (2009) found that one session of peripheral nerve stimulation at the intensity of strong paresthesia, but with no visible motor movement or pain, and a frequency of 10 Hz and duration of 2 h, resulted in improvement in pinch strength in the paretic hand of stroke patients. Nito et al. (2021) showed that after a single session of rPMS at a frequency of 25 and 50 Hz and intensity of 120% of resting motor threshold resulted in an increase in wrist extension force and EMG activity during ballistic wrist extension movements. Struppler et al. (1996) investigated the effect of rPMS in paretic hands in stroke patients and found that patients reported an improvement in grasping and finger extension movement after two rPMS sessions, and that the improvement in movement outlasted the stimulation period by a number of days. The authors proposed that rPMS may increase proprioceptive input *via* the lemniscal-thalamic pathways projecting to the sensorimotor cortex and modulating corticospinal motor command, resulting in increased cortical excitability and improvement in motor performance (Struppler et al., 1996; Nito et al., 2021). Between-group comparison showed that the rPMS group had an improvement of pinch strength of 1.9 lbs over the control group with the 95% confidence interval [−0.3 to 4.1] (*p* = 0.09). Although not statistically significant in our pilot study, rPMS may help increase pinch strength and this warrants further study (Sullivan & Feinn, 2012).

After rPMS treatment, there was a significant improvement in symptom severity scores (SSS) as measured by the Thai version of the Boston Questionnaire. In accordance with the findings of Khedr et al. (2012), muscle strength and pain began to improve in participants with traumatic brachial plexus injury following five sessions of rPMS. El Gohary et al. (2015) demonstrated that three sessions of rPMS significantly alleviated pain in patients with early CTS.

Although the improvement in DSL in the rPMS group was not significant, the increase in SNAP amplitude was. Previous research on CTS treatment trended to concentrate on distal motor and sensory latency (DML and DSL) parameters, but not SNAP amplitude. For instance, a recent meta-analysis revealed that therapeutic ultrasound in CTS significantly improved DML with a mean effect size of 0.09 ms (Peris Moya et al., 2022), but had no effect on DSL. SNAP or CMAP amplitude was not typically discussed, which may be because the primary diagnostic criteria for CTS centered on DML and DSL, and the improvement in these parameters can indicate a change in CTS severity. Ashraf et al. (2020) reported that SNAP appeared in 63% of patients with severe CTS after an initial absence of SNAP following a 1-year follow-up electrodiagnostic study. Furthermore, in patients with low SNAP findings, SNAP amplitude and sensory latency at the wrist showed significant improvement (Ashraf et al., 2020). Therefore, we suggest that the increase in SNAP amplitude in this study may be indicative of an early neurophysiological improvement; however, this requires further investigation. Caution should also be applied as it was found that the amplitude of SNAPs were rather variable (Diozzi et al., 2017).

Although there was no significant difference between the severity category of the two treatment groups, the rPMS group appeared to have better results than the control group, as two out of 12 participants (16.7%) changed from mild or moderate CTS to normal electrophysiologic findings. If the conversion rate remains high in a study with a larger sample size, rPMS would be a promising treatment for CTS.

This research has several limitations. First, the duration of treatment was brief, so the long-term effect is unknown. Second, patients in the rPMS group reported improved hand pain *via* personal communication. Multiple studies demonstrate that rPMS can alleviate neuropathic pain, indicating that rPMS may provide immediate analgesic effects, and repeated rPMS sessions may augment this effect (El Gohary et al., 2015; Lefaucheur, Drouot & Nguyen, 2001). In addition to recording SSS of the Thai version of the Boston Questionnaire (Th-BS SSS), which has questions related to pain, some specific pain quantification such as numeric rating scale should be incorporated as an outcome measure in future research. Third, we did not use a sham control but instead used our hospital’s standard usual care. While this may reduce the internal validity of the RCT, it does reflect real-world practice, thus providing information for deciding whether PMS adds additional benefits to conventional management in patients with CTS. Lastly, a larger sample size should be considered in future studies. In addition, future research should employ rPMS with alternating protocols to determine the optimal therapeutic parameters. This study’s findings was restricted to mild to moderate degrees of CTS and cannot be generalized to severe CTS.

## Conclusions

Five sessions of rPMS resulted in significant improvements in symptom severity, pinch strength, and neurophysiological parameters; however, there was no significant positive effect of rPMS when comparing all outcomes between the two groups. Future research should investigate the clinical utility of rPMS using a larger population sample and a longer treatment and follow-up.

## Supplemental Information

10.7717/peerj.15398/supp-1 Supplemental Information 1 Raw data.

10.7717/peerj.15398/supp-2 Supplemental Information 2 Consort checklist.

10.7717/peerj.15398/supp-3 Supplemental Information 3 Trial registry number and protocol.

## Additional Information and Declarations

### Data Availability

The following information was supplied regarding data availability: The raw data are available in the Supplemental Files.

### Clinical Trial Registration

The following information was supplied regarding Clinical Trial registration: TCTR 20200828002
