# Supplementary material for: Effects of repetitive peripheral magnetic stimulation vs. conventional therapy in the management of carpal tunnel syndrome: a pilot randomized controlled trial
Source: PeerJ. 2023 May 18;11:e15398. doi: 10.7717/peerj.15398 (PMC10200096; doi:10.7717/peerj.15398)
Supplement: Supplemental Information 3 [file peerj-11-15398-s003.pdf]

TCTR ID : TCTR20200828002

OTHER ID :

Overall Recruitment Status : Completed **(Has Results)**

Prospective registration
This protocol was registered before enrollment of the first participant.

Tracking Information

First Submitted Date : 23 August 2020

First Posted Date : 28 August 2020

Last Update Posted Date : 11 November 2022

Title

Public Title : Comparison of the effectiveness between Peripheral Repetitive Magnetic Stimulation and Conventional Therapy in Carpal Tunnel Syndrome: a pilot randomized controlled trial

Acronym : RPMS

Scientific Title : Comparison of the effectiveness between Peripheral Repetitive Magnetic Stimulation and Conventional Therapy in Carpal Tunnel Syndrome: a pilot randomized controlled trial

Sponsor ID/ IRB ID/ EC ID : HE621319

Registration Site : Thai Clinical Trials Registry

URL : https://www.thaiclinicaltrials.org/show/TCTR20200828002

Secondary ID : No Secondary ID

Ethics Review

1. Board Approval : Submitted, approved

Approval Number : HE621319

Date of Approval : No Data

Board Name : Khon Kaen University Ethics Committee of Human Research

Board Affiliation : Khon Kaen University, Thailand

Board Contact : Business Phone : +66+43+366616 Ext. -

Business Email : eckku@kku.acth

Business Address : Khon Kaen University Ethics Committee of Human Research 123 Friendship Road Khon Kaen University, 40002 Thailand

Sponsor

Source(s) of Monetary or Material Supports : Khon Kaen University, faculty of medicine

Study Primary Sponsor : General Research Grant

Responsible Party : Name/Official Title : Faculty of medicine

Organization : General Research Grant

Phone : 043348360 Ext. No Data

Email : sureesri289@gmail.com

Study Secondary Sponsor : No Study Secondary Sponsor

Protocol Synopsis

Protocol Synopsis : Peripheral nerve repetitive magnetic stimulation is a new physical agent that can be used to relieve pain and to recover hand function. The aim of this study was to evaluate the therapeutic effects of peripheral repetitive magnetic stimulation in carpal
tunnel syndrome.Carpal tunnel syndrome is the most common entrapment neuropathy in the upper extremities. In non-severe case, conservative treatment is the wide range theraputic options. Peripheral nerve repetitive magnetic stimulation is a new noninvasive physical agent that can be used as conservative treatment in non-severe carpal tunnel syndrome. We will investigate the effect of Peripheral nerve repetitive magnetic stimulation compared with conservative treatment on impoving symptom and hand function in mild to moderate carpal tunnel syndrome. The outcome-measuring tool are Thai version Boston Questionaire to evaluate severity of symptoms and the degree of manual skill, nerve conduction study and pinch strength.

URL not available

Health Conditions

Health Condition(s) or Problem(s) Studied : severe degree of carpal tunnel syndrome

Keywords : carpal tunnel syndrome Peripheral Repetitive Magnetic Stimulation

Eligibility

Inclusion Criteria : The study included patients with age above 18
years with electrophysiological proof of having carpal tunnel syndrome (mild to moderate degree)

Gender : Both

Age Limit : Minimum : 18 Years Maximum : N/A (No limit)

Exclusion Criteria : 1.Patients with history of surgical release of carpal tunnel
2.Patients with previous wrist fracture or wrist dislocation
3.Patients with underlying disease such as diabetes mellitus, hyperthyroidism and hypothyroidism and rheumatoid arthritis.
4.Patients with history suggestive of any neurological disease such as polyneuropathy, cervical radiculopathy, ulnar neuropathy
5.Patients with magnetic Stimulate contraindication such as installing pacemakers, cochlear implant or intracranial metal objects, pregnant females or those who are not consenting for the procedures.

Accept Healthy Volunteers : No

Status

Overall Recruitment Status : Completed

Key Trial Dates Study Start Date (First enrollment) : 01 September 2020 Indicate Type : Actual

Completion Date (Last subject, Last visit) : 11 January 2021 Indicate Type : Actual

Study Completion Date : 25 January 2021 Indicate Type : Actual

Design

Study Type : Interventional

Primary Purpose : Treatment

Study Phase : Phase 2

Intervention Model : Parallel

Number of Arms : 2

Masking :

Allocation : Randomized

Control : Placebo

Study Endpoint Classification : Safety/Efficacy Study

Sample size

Planned sample size : 24

Actual sample size at study completion :

Intervantion Arm 1

Intervention name : Repetitive Peripheral Magnetic Stimulation

Intervention Type : Experimental

Intervention Classification : Device

Intervention Description : Paticipant will have 5 sessions of Peripheral Repetitive Magnetic Stimulation with the coil located at the wrist. The magnetic stimulation protocol consisted in 100 trains of 10 pulses/train, delivered at 10 Hz

Intervantion Arm 2

Intervention name : Conventional Therapy

Intervention Type : No Intervention

Intervention Classification : Behavioral

Intervention Description : Tendon Gliding exercises and avoidance of wrist postures (i.e., prolonged wrist flexion) or repetitive wrist motions

Outcome

Primary Outcome

1. Outcome Name : symptom and functional severity

Metric / Method of measurement : Thai Version Boston Questionnaire

Time point : 2 weeks after end of the intervention

Secondary Outcome

1. Outcome Name : pinch strength

Metric / Method of measurement : using pinch gauges to measure strenght

Time point : 2 weeks after end of the intervention

Location

Section A : Central Contact

Central Contact	First Name : Arachaphon	Middle Name :	Last Name : Panathoop
	Degree : MD	Phone : 0650564147 Ext. : No Data	Email : aracha818@gmail.com
Central Contact Backup	First Name : Ratana	Middle Name :	Lastname : Vichiansiri
	Degree : MD	Phone : 0810525610 Ext. : No Data	Email : vratana@kku.ac.th

Section B Facility Information and Contact

1.	Site Name : Faculty of Medicine		
	City : Mueang Khon Kaen	State/Province : Khon Kaen	Postal Code : 40000
	Country : Thailand	Recruitment Status : Pending (Not yet recruiting)	
**Facility Contact**	First Name : Arachaphon	Middle Name :	Last Name : Panathoop
	Degree : MD	Phone : 0987502218 Ext. : No Data	Email : aracha818@gmail.com
**Facility Contact Backup**	First Name : Ratana	Middle Name :	Last Name : Vichiansiri
	Degree : MD	Phone : +66 43-348392 Ext. : No Data	Email : vratana@kku.ac.th
**Investigator Name**	First Name : Arachaphon	Middle Name :	Last Name : Panathoop
	Degree : MD	Role : Principal Investigator	

Section C : Contact for Public Queries (Responsible Person)

First Name : Arachaphon	Middle Name :	Last Name : Panathoop
Degree : MD	Phone : 0650564147 Ext. : No Data	Email : aracha818@gmail.com
Postal Address : 123 Mittraphap Rd, Mueang Khon Kaen District		
State/Province : Khon kaen	Postal Code : 40000	
Country : Thailand	Official Role : Study Principal Investigator	
Organization Affiliation : Faculty of medicine, Khon Kean University		

Section D : Contact for Scientific Queries (Responsible Person)

First Name : Arachaphon	Middle Name :	Last Name : Panathoop
Degree : MD	Phone : 0650564147 Ext. : No Data	Email : aracha818@gmail.com
Postal Address : 123 Mittraphap Rd, Mueang Khon Kaen District		
State/Province : Khon kaen	Postal Code : 40000	
Country : Thailand	Official Role : Study Principal Investigator	
Organization Affiliation : Faculty of medicine, Khon Kean University		

Summary Results

Date of posting of results summaries : 27 October 2022

Date of first journal publication of results : Not yet published

Baseline Characteristics : In rPMS group, their average age was 49.3 (SD 10.8) years. Most of them were female (91.7%). The average age in control group was 52.3 (SD 7.1) years and 66.7 % were female. Median duration of working time in rPMS and control were 8 hours/day, 7 hours /day respectively. Median duration of CTS symptom in rPMS group was 4 months and in control group was 3.5 months

Participant Flow : A total of 24 participants were enrolled in this study. There were divided into 2 groups of 12 equal participants each.

Adverse events : No unfavorable change in the health of a participant and all serious adverse events

Outcome Measures : Only rPMS group showed significant improvement in the 2 week-outcome including symptom severity scores (SSS) determined by Thai version Boston Questionnaireand pinch strength. For the nerve conduction study, only rPMS showed a significant increment in SNAP amplitude of median nerve. Although some improvements were found in symptom severity score and SNAP amplitude but these did not reach statistically significant level. There was no statistically significant difference when compared all final outcomes between two groups

Brief Summary of Results : The rPMS group showed significantly greater within group improvement in symptom severity scores and pinch strength. Regarding electrodiagnostic parameters, sensory nerve action potential amplitude was significantly increased within group treated with rPMS. There was no statistically significant within group difference in conventional therapy. Although overall changes in PMS group were of greater magnitude, no statistically significant difference was found when comparing between groups.

Deidentified Individual Participant-level Data Sharing

Plan to share IPD : Yes

Plan description : European Journal of Physical and Rehabilitation Medicine and registered under number Eur J Phys Rehabil Med-7768

Publication from this study

MEDLINE Identifier : No Data

URL link to full text publication : No Data

This document was created on 11 November 2022 at 14:56 (GMT+7).
